# Supplementary material for: Zebrafish modeling reveals that SPINT1 regulates the aggressiveness of skin cutaneous melanoma and its crosstalk with tumor immune microenvironment
Source: J Exp Clin Cancer Res. 2019 Sep 13;38:405. doi: 10.1186/s13046-019-1389-3 (PMC6743187; doi:10.1186/s13046-019-1389-3)
Supplement: Supplementary file 1 — Figure S1. Spint1a-deficient SKCM shows enhanced aggressiveness in adult zebrafish allotransplantation assays. Control and Spint1a deficient SKCMs were disaggregated and 30,000 (a), 100,000 (b) and 300,000 cells (c) were injected subcutaneously in pre-irradiated adult casper zebrafish. Fish were analyzed for average tumor size (pixels) from 1 to 4 weeks post-transplant. Representative images and quantification of the average tumor size are shown. Each dot corresponds to a recipient-transplanted fish and the mean ± SEM is also shown (n=2 SKCM tumors).*p<0.05, ***p<0.001 according to unpaired Student t test. Figure S2. Expression analysis of differentiation melanocyte, EMT, inflammation and immune cell markers in zebrafish SKCM. The mRNA levels of the genes encoding the differentiation melanocyte markers sox10, mitfa, tyr and dct, the EMT markers cdh1, slug and mmp9, the inflammation marker il1b, the neutrophil markers lyz and mpx, the macrophage marker mpeg1, and the ISGs b2m, mxb and pkz were analyzed by RT-qPCR in control and Spint1a-deficient SKCMs. *p < 0.05, **p<0.01 according to one-tailed Student t test. Table S1. Primers used in this study for RT-qPCR. The gene symbols followed the Zebrafish Nomenclature Guidelines (http://zfin.org/zf_info/nomen.html/). ENA, European Nucleotide Archive (http://www.ebi.ac.uk/ena/). (PDF 496 kb) [file 13046_2019_1389_MOESM1_ESM.pdf]

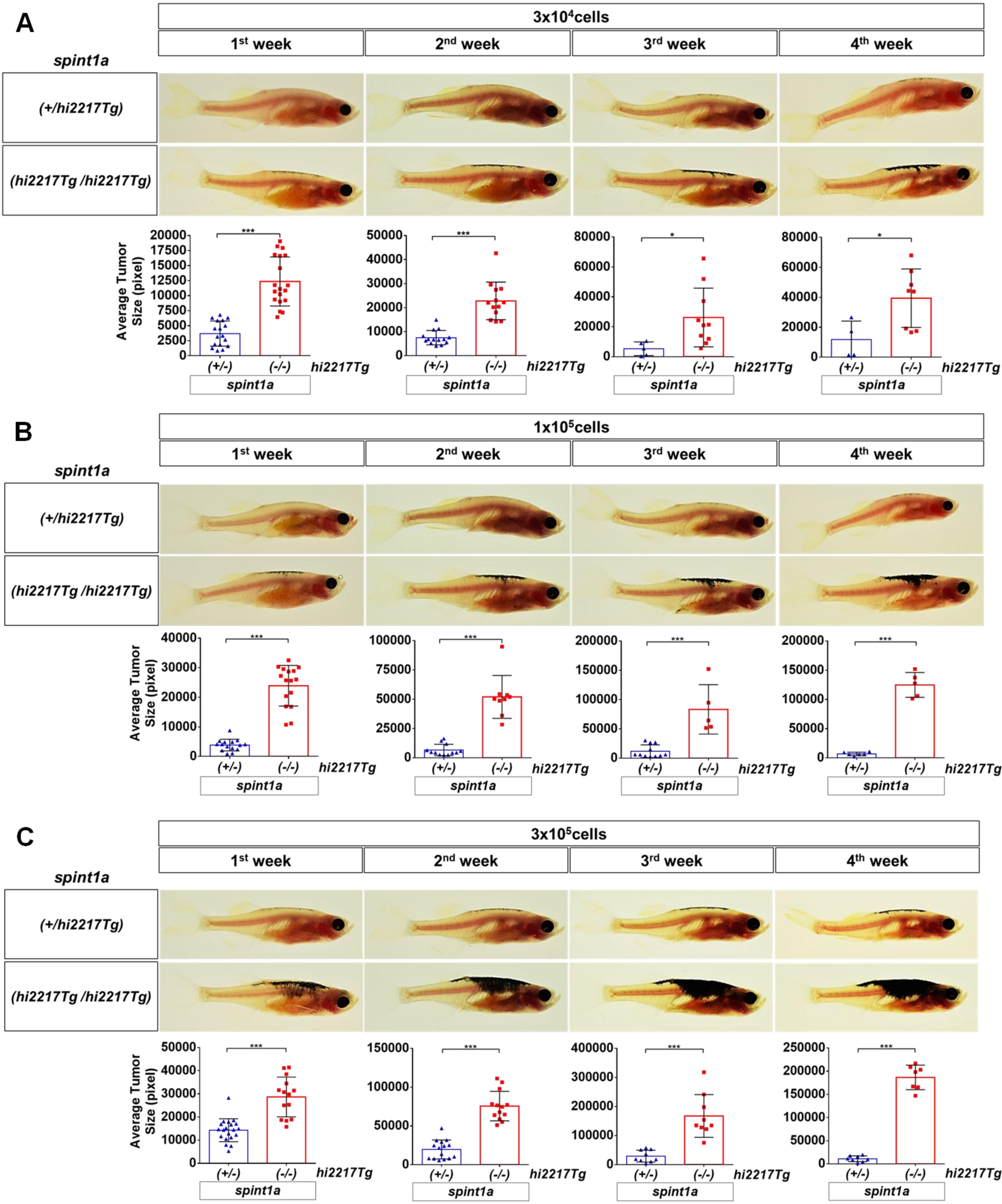

Figure S1. Spint1a-deficient SKCM shows enhanced aggressiveness in adult zebrafish allotransplantation assays. Control and Spint1a deficient SKCMs were disaggregated and 30,000 **(A)**, 100,000 **(B)** and 300,000 cells **(C)** were injected subcutaneously in pre-irradiated adult casper zebrafish. Fish were analyzed for average tumor size (pixels) from 1 to 4 weeks post-transplant. Representative images and quantification of the average tumor size are shown. Each dot corresponds to a recipient-transplanted fish and the mean ± SEM is also shown (n=2 SKCM tumors).*$p<0.05$, ***$p<0.001$ according to unpaired Student *t* test.

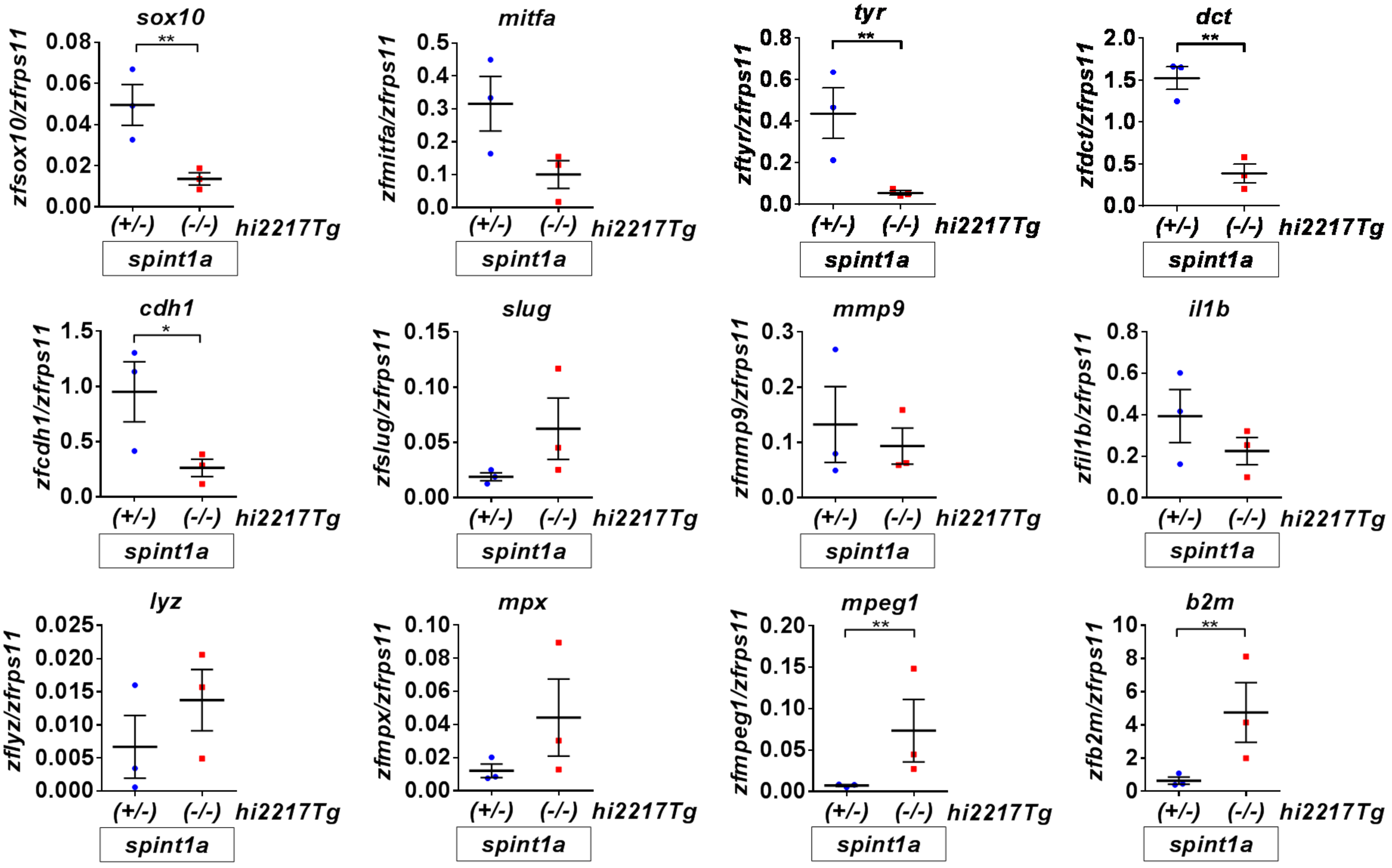

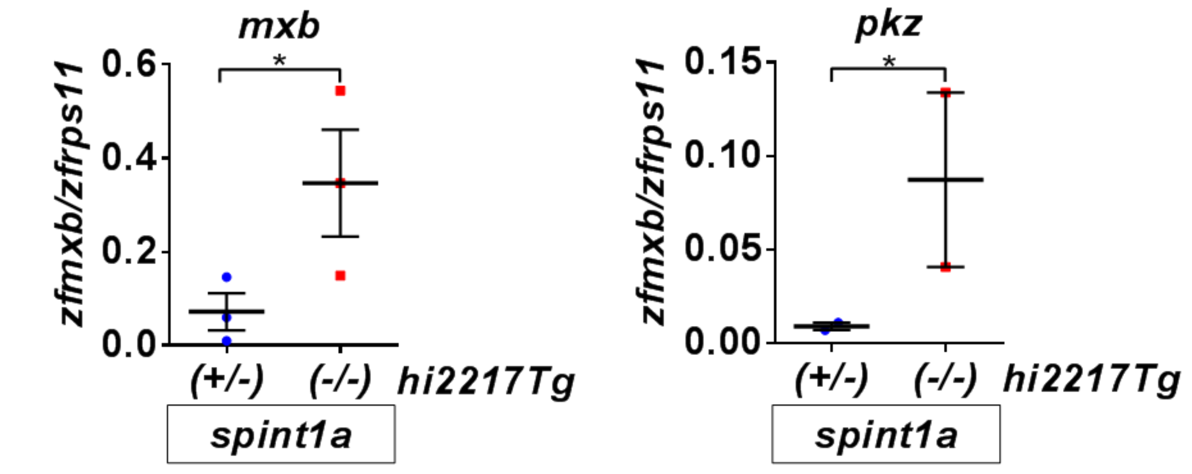

Figure S2. Expression analysis of differentiation melanocyte, EMT, inflammation and immune cell markers in zebrafish SKCM. The mRNA levels of the genes encoding the differentiation melanocyte markers *Sox10*, *Mitfa*, *Tyr* and *Dct*, the EMT markers *Cdh1*, *Slug* and *Mmp9*, the inflammation marker *Il1b*, the neutrophil markers *Lyz* and *Mpx*, the macrophage marker *Mpeg1*, and the ISGs *B2m*, *Mxb* and *Pkz* were analyzed by RT-qPCR in control and Spint1a-deficient SKCMs. $*p < 0.05$, $**p<0.01$ according to one-tailed Student *t* test.

Table S1. Primers used in this study for RT-qPCR. The gene symbols followed the Zebrafish Nomenclature Guidelines (http://zfin.org/zf_info/nomen.html/). ENA, European Nucleotide Archive (http://www.ebi.ac.uk/ena/).

Gene	ENA ID	Name	Sequence (5’→3’)
rps11	NM_213377	F1	GGCGTCAACGTGTCAGAGTA
		R1	GCCTCTTCTCAAAACGGTTG
sox10	NM_131875.1	F	CCTCACGCTACAGGTCAGAG
		R	CGAAGTCGATGTGCGGTTTC
mitfa	NM_001362262.1	F	CGACTGGTCAGTTCTTGCAC
		R	AGGTGGGTCTGAACCTGGTA
tyr	NM_131013.3	F	TGTATTCATGAACGGCTCCA
		R	GATGAAGGGCACCATGAAGT
dct	NM_131555.2	F	TGGACAGTAAACCCTGGGGA
		R	CCGGCAAAGTTTCCAAAGCA
cdh1	NM_131820.1	F	TGGCAAAAGACTAGGCAAAGTGAC
		R	AAACACCTTGTGGCCCTCAT
slug	NM_001008581.1	F1	AGTCCAACAGTGTTTATTTCTCCA
		R1	GCAGGTTGCTGGTAGTCCAT
mmp9	NM_213123.1	F1	GCTGCTCATGAGTTTGGACA
		R1	AGGGCCAGTTCTAGGTCCAT
il1b	NM_212844.2	F5	GGCTGTGTGTTTGGGAATCT
		R5	TGATAAACCAACCGGGACA
lyz	NM_139180.1	F	TGGCAGTGGTGTTTTTGTGT
		R	TCAAATCCATCAAGCCCTTC
mpx	NM_212779	F1	AGGGCGTGACCATGCTATAC
		R1	AGGCTCAGCAACACCTCCTA
mpeg1	NM_212737.1	F	ACAGCAAAACACCCATCTGGCGA
		R	TGCGGCACAATCGCAGTCCA
b2m	NM_001159768.1	F	AACCAAACACCCTGATCTGC
		R	CAACGCTCTTTGTGAGGTGA
mxb	NM_001128672.1	F	AATGGTGATCCGCTATCTGC
		R	TCTGGCGGCTCAGTAAGTTT
pkz	NM_001040376.2	F1	GGAGCACCGTACAGGACATT
		R1	CTCGGGCTTTATTTGCTCTG
